# Supplementary material for: Risk Factors for Exposure of Wild Birds to West Nile Virus in A Gradient of Wildlife-Livestock Interaction
Source: Pathogens. 2023 Jan 3;12(1):83. doi: 10.3390/pathogens12010083 (PMC9864363; doi:10.3390/pathogens12010083)
Supplement: Supplementary file 1 [file pathogens-12-00083-s001.zip › pathogens-2127866-supplementary.pdf]

Table S1. Results of the scientific literature survey of selected studies that used the INGEZIM West Nile COMPAC blocking ELISA (bELISA; Ingenasa, Spain) and performed viral neutralization tests (VNT) against different *Flavivirus* spp. (at least WNV and USUV). The target animal group, the spatial range of surveyed animals and the country where the animals came from are together shown with the number of bELISA analysed samples to show the extent of the study. Studies performed in wild birds are marked in bold letter type.

Target animal group	Spatial range	Country	No. bELISA tested	No. VNT-confirmed *Flavivirus* spp.	No. VNT-confirmed WNV	No. VNT-confirmed other *Flavivirus* spp.	WNV to other *Flavivirus* spp. predominance ratio	Reference
**Feral pigeons**	**Córdoba city**	**Spain**	**142**	**6**	**3**	**3**[a]	**50%**	[42]
**Wild birds**	**Country**	**Poland**	**474**	**63**	**63**	**0**	**100%**	[43]
**Wild birds**	**South-western Spain**	**Spain**	**149**	**2**	**1**	**1**	**50%**	[44]
Wild ruminants	Country	Spain	4693	109	103	6[b]	94.5%	[45]
**Wild raptors**	**Andalusia**	**Spain**	**123**	**4**	**4**	**0**	**100%**	[46]
Domestic dogs	South-western & west-central Spain	Spain	815	25	11	14[c]	44%	[47]
Passer domesticus	**South-western Spain**	**Spain**	**2544**	**18**	**17**	**1**[a]	**94.4%**	[48]
Zoo mammals	Country	Spain	570	16	10	6[d]	62.5%	[49]
Horses	West-central Spain	Spain	725	154	143	11[a]	92.9%	[5]
**Wild birds**	**West-central Spain**	**Spain**	**384**	**74**	**70**	**4**[a]	**94.6%**	[36]
**Wild birds**	**Badajoz city vicinity**	**Spain**	**645**	**21**	**20**	**1**[a]	**95.2%**	[37]

[a]VNT-confirmed as USUV antibodies; [b]VNT-confirmed as USUV (n=4) and Meaban virus (n=1) antibodies; [c]VNT-confirmed as tick-borne encephalitis virus (TBEV) antibodies; [d]VNT-confirmed as USUV (n=5) and TBEV (n=1) antibodies.

Table S2. Total number of captured mosquitoes by genus, study site and interaction gradient in the five study sites. Specific capture data for the two predominant mosquito species, *Culex pipiens* and *Cx. theileri* is shown.

Site	Area	*Culex pipiens*	*Culex theileri*	*Culex* spp.	*Cs.** spp.	*An.** spp.	*Ae.** spp.	Undt.*	Others	TOTAL
	A_1	33	17	51	0	0	1	1	0	53
S_1	A_2	117	63	180	1	3	4	12	0	200
	A_3	2	3	5	0	0	0	2	0	7
Subtotal S_1		152	83	236	1	3	5	15	0	260
	A_1	19	61	80	0	14	0	3	0	97
S_2	A_2	23	29	52	1	7	2	2	0	64
	A_3	36	41	77	3	3	3	10	0	96
Subtotal S_2		78	131	209	4	24	5	15	0	257
	A_1	77	35	112	3	1	12	3	0	131
S_3	A_2	34	5	39	1	0	1	1	0	42
	A_3	22	3	26	0	1	6	5	0	38
Subtotal S_3		133	43	177	4	2	19	9	0	211
	A_1	1	17	18	1	0	8	3	0	30
S_4	A_2	14	0	14	0	0	2	2	0	18
	A_3	28	15	43	2	3	21	2	0	71
Subtotal S_4		43	32	75	3	3	31	7	0	119
	A_1	70	66	138	7	3	16	7	0	171
S_5	A_2	171	30	202	3	6	21	11	0	243
	A_3	212	100	312	8	5	23	19	1	368
Subtotal S_5		453	196	652	18	14	60	37	1	782
	A_1	200	196	399	11	18	37	17	0	482
All	A_2	359	127	487	6	16	30	28	0	567
	A_3	300	162	463	13	12	53	38	1	580
Total		859	485	1349	30	46	120	83	1	1629

*Cs.: *Culiseta* spp.; An.: *Anopheles* spp.; Ae.: *Aedes* spp.; Undt.: Undetermined genus.

Table S3. Number of birds caught per site (S_1-S_5), area (A_1-A_3) and family and results of blocking ELISA (serum) and rRT-PCR (feather cannon pulp, oral or cloacal swab).

Site	Area	Bird family	No. captures	bELISA No. tested /No. positive	rRT-PCR No. tested /No. positive	bELISA No. tested /No. positive (Prevalence)	rRT-PCR No. tested /No. positive (Prevalence)
S_1	A_1	*Corvidae*	10	10/1	10/0	34/4 (12.9%)	33/0 (0%)
		Emberizidae	1	1/0	1/0		
		Fringilidae	3	3/0	1/0		
		Fringillidae	4	3/0	3/0		
		Hirundidae	1	1/0	1/0		
		Paridae	5	5/3	5/0		
		Passeridae	7	7/0	6/0		
		Phylloscopidae	1	0/0	1/0		
		Sittidae	1	1/0	1/0		
		Sylviidae	1	1/0	1/0		
		Turdidae	1	1/0	1/0		
		Upupidae	1	1/0	1/0		
	A_2	*Acrocephalidae*	2	0/0	2/0	7/0 (0%)	28/0 (0%)
		Cettidae	3	0/0	3/0		
		Cisticolidae	1	0/0	1/0		
		Emberizidae	3	0/0	3/0		
		Fringilidae	1	1/0	1/0		
		Hirundidae	2	0/0	2/0		
		Laniidae	3	3/0	3/0		
		Paridae	3	1/0	3/0		
		Passeridae	1	1/0	1/0		
		Sturnidae	1	1/0	1/0		
		Sylviidae	8	0/0	8/0		
	A_3	*Acrocephalidae*	1	0/0	1/0	20/2 (10%)	33/0 (0%)
		Aegithalidae	3	0/0	3/0		
		Corvidae	5	5/0	5/0		
		Motacillidae	1	1/0	1/0		
		Muscicapidae	1	1/0	1/0		
		Paridae	14	6/1	14/0		
		Phylloscopidae	1	1/0	1/0		
		Sittidae	1	1/0	1/0		
		Sturnidae	1	1/0	1/0		
		Sylviidae	3	1/0	3/0		
		Turdidae	3	3/1	2/0		
S_2	A_1	*Fringillidae*	4	3/1	4/0	48/2 (4.2%)	43/0 (0%)
		Hirundidae	8	8/0	6/0		
		Laniidae	4	4/0	4/0		
		Paridae	9	4/0	8/0		
		Passeridae	19	18/0	14/0		
		Sturnidae	2	2/0	2/0		
		Sylviidae	4	3/0	2/0		
		Turdidae	6	6/1	3/0		
	A_2	*Aegithalidae*	5	1/0	5/0	44/2 (4.5%)	58/1 (1.7%)
		Corvidae	5	5/0	5/0		

		Emberizidae	1	1/0	1/0		
		Paridae	28	15/1	25/0		
		Phylloscopidae	3	2/0	1/0		
		Sturnidae	1	1/0	1/0		
		Sylviidae	7	3/0	7/0		
		Turdidae	17	16/1	13/1		
	A_3	*Corvidae*	6	6/0	5/0	27/0 (0%)	34/0 (0%)
		Emberizidae	2	2/0	2/0		
		Hirundidae	1	1/0	0/0		
		Laniidae	2	1/0	2/0		
		Paridae	22	14/0	22/0		
		Sylviidae	3	1/0	2/0		
		Turdidae	2	2/0	1/0		
S_3	A_1	*Emberizidae*	2	2/0	2/0	18/3 (16.7%)	37/1 (2.7%)
		Fringillidae	6	3/2	4/0		
		Muscicapidae	1	1/0	1/0		
		Oriolidae	1	1/0	1/0		
		Paridae	7	4/0	7/0		
		Phylloscopidae	1	1/0	1/0		
		Sylviidae	2	2/0	2/0		
		Turdidae	4	4/1	4/1		
	A_2	*Aegithalidae*	2	1/0	2/0	32/1 (3.1%)	37/1 (2.7%)
		Corvidae	1	1/0	1/0		
		Emberizidae	12	7/1	10/1		
		Fringillidae	5	5/0	3/0		
		Muscicapidae	3	2/0	3/0		
		Paridae	14	10/0	10/0		
		Passeridae	3	3/0	3/0		
		Sittidae	1	0/0	1/0		
		Sylviidae	2	1/0	2/0		
		Turdidae	2	2/0	2/0		
	A_3	*Aegithalidae*	4	2/0	3/0	32/0 (0%)	32/0 (0%)
		Cettidae	1	1/0	1/0		
		Emberizidae	1	0/0	0/0		
		Fringillidae	4	3/0	4/0		
		Laniidae	2	2/0	1/0		
		Muscicapidae	4	4/0	2/0		
		Paridae	8	7/0	8/0		
		Passeridae	4	3/0	4/0		
		Phylloscopidae	1	1/0	1/0		
		Prunellidae	1	1/0	1/0		
		Sylviidae	5	5/0	4/0		
		Turdidae	3	3/0	3/0		
S_4	A_1	*Paridae*	5	5/1	5/0	23/3 (13%)	22/0 (0%)
		Passeridae	17	16/2	15/0		
		Sittidae	2	2/0	2/0		
	A_2	*Columbidae*	15	15/0	14/0	25/1 (4%)	24/0 (0%)
		Passeridae	10	10/1	10/0		
		Aegithalidae	6	0/0	6/0		
	A_3	*Certhiidae*	2	2/0	2/0	34/1 (2.9%)	45/0 (0%)
		Fringillidae	5	5/0	5/0		
		Muscicapidae	6	6/0	6/0		
		Paridae	26	21/1	26/0		

S_5	A_1	*Hirundidae*	8	7/0	7/0	32/2 (6.3%)	32/0 (0%)
		Paridae	1	1/0	1/0		
		Passeridae	25	24/2	24/0		
	A_2	*Aegithalidae*	1	1/0	1/0	35/4 (11.4%)	30/1 (3.3%)
		Alcedinidae	1	1/0	1/0		
		Certhiidae	3	3/0	3/0		
		Cettidae	1	0/0	0/0		
		Corvidae	1	1/0	1/0		
		Fringillidae	2	1/0	0/0		
		Muscicapidae	1	1/0	1/1		
		Paridae	16	15/2	15/0		
		Phylloscopidae	7	7/0	3/0		
		Sylviidae	2	2/0	2/0		
		Turdidae	3	3/2	3/0		
	A_3	*Certhiidae*	1	1/0	1/0	25/5 (20%)	26/0 (0%)
		Corvidae	2	2/0	1/0		
		Laniidae	1	1/0	1/0		
		Paridae	18	17/5	18/0		
		Sylviidae	7	5/0	5/0		

Table S4. Bird abundance indices per site and wildlife-livestock interaction scenario throughout bird taxonomic Order and Family. Within each taxonomic level, the highest abundance index of the three interaction scenarios is shown in bolded type letter case.

			**S_1**			**S_2**			**S_3**			**S_4**			**S_5**			**S_1-S_5**	
**Order**	**Family**	**A_1**	**A_2**	**A_3**	**A_1**	**A_2**	**A_3**	**A_1**	**A_2**	**A_3**	**A_1**	**A_2**	**A_3**	**A_1**	**A_2**	**A_3**	**A_1**	**A_2**	**A_3**
	Passeridae	**2.583**	0.250	0.000	**14.333**	0.000	3.188	0.250	0.000	**0.813**	**4.583**	1.000	0.313	**22.750**	10.250	1.771	**8.900**	2.300	1.217
	Hirundinidae	**0.917**	0.500	0.250	0.354	0.125	**0.417**	0.000	0.000	0.000	**0.417**	0.083	0.000	3.396	**3.458**	0.229	**1.017**	0.833	0.179
	Lanidae	**0.979**	0.563	0.500	0.542	0.875	**1.188**	0.063	0.000	**0.333**	**0.938**	0.333	0.208	3.396	**6.083**	0.542	1.183	**1.571**	0.554
	Alaudidae	0.271	**0.875**	0.083	0.646	0.146	**0.896**	**0.896**	0.479	0.063	**0.688**	0.208	0.625	0.000	0.000	0.000	**0.500**	0.342	0.333
	Aegithalidae	0.250	0.063	**0.583**	0.000	0.000	0.000	**0.250**	0.000	0.125	0.000	0.083	**0.417**	0.000	**3.042**	0.125	0.100	**0.638**	0.250
	Emberizidae	**1.167**	1.042	0.667	0.313	0.000	**0.896**	**0.938**	0.875	0.000	**4.250**	0.229	0.813	**1.667**	0.000	0.000	**1.667**	0.429	0.475
	Motacillidae	0.000	0.000	0.000	0.000	0.000	0.000	0.000	0.000	0.000	0.000	0.000	0.000	**0.458**	0.000	0.083	**0.092**	0.000	0.017
	Turdidae	0.167	0.125	**0.271**	0.000	**0.958**	0.000	0.292	0.125	**0.604**	0.000	0.000	**0.229**	0.083	**0.625**	0.063	0.108	**0.367**	0.233
	Paridae	0.938	**1.375**	0.729	0.146	**0.188**	0.083	**1.188**	1.021	**1.188**	0.542	0.313	**1.417**	0.146	**2.583**	0.625	0.592	**1.096**	0.808
	Sturnidae	1.375	**1.792**	0.063	1.813	**3.583**	0.000	**3.229**	0.708	0.000	0.188	0.438	**1.729**	83.313	**137.500**	2.083	17.983	**28.804**	0.775
**Passeriformes**	*Sylviidae*	0.063	**0.125**	**0.125**	0.063	**0.292**	0.083	0.000	**0.292**	0.083	0.000	0.063	**0.146**	0.000	0.458	**0.729**	0.025	**0.246**	0.233
	Corvidae	**0.333**	0.188	0.167	1.313	**8.833**	3.458	0.438	0.125	**0.979**	0.063	0.000	**0.188**	0.500	**2.750**	1.896	0.529	**2.379**	1.338
	Cisticolidae	0.000	0.000	0.000	0.000	0.000	0.000	**0.083**	0.000	0.000	0.000	0.000	0.000	0.000	0.000	**0.063**	**0.017**	0.000	0.013
	Fringilliidae	0.729	**0.771**	0.292	0.229	0.229	**0.688**	0.458	0.854	**5.500**	1.313	0.313	**1.438**	**0.583**	0.375	0.313	0.663	0.508	**1.646**
	Sittidae	0.000	0.000	**0.063**	0.000	0.000	0.000	0.167	0.375	**0.438**	**0.125**	0.083	0.083	0.000	0.000	0.000	0.058	0.092	**0.117**
	Muscicapidae	0.000	**0.292**	0.000	0.000	0.000	0.000	0.000	**0.083**	**0.083**	0.000	**0.417**	0.292	**0.083**	0.000	**0.083**	0.017	**0.158**	0.092
	Cettidae	**0.208**	0.146	0.000	0.000	0.000	0.000	0.000	0.000	0.000	0.000	0.000	0.000	0.000	0.000	**0.083**	**0.042**	0.029	0.017
	Oriolidae	0.000	0.083	**0.229**	0.000	0.000	0.000	0.229	**0.542**	0.000	0.000	0.000	0.000	0.000	0.000	0.000	0.046	**0.125**	0.046
	Acrocephalidae	0.000	**0.313**	0.000	0.000	0.000	0.000	0.000	0.000	0.000	0.000	0.000	0.000	**0.083**	0.000	0.000	0.017	**0.063**	0.000
	Phylloscopidae	0.000	0.000	0.000	0.000	0.000	0.000	0.000	0.000	0.000	0.000	0.000	0.000	0.000	0.000	0.000	0.000	0.000	0.000
	Subtotal	***9.979***	*8.500*	*4.021*	***19.750***	*15.229*	*10.896*	*8.479*	*5.479*	***10.208***	***13.104***	*3.563*	*7.896*	*116.458*	***167.125***	*8.688*	***33.554***	*39.979*	*8.342*
**Columbiformes**	*Columbidae*	**2.083**	1.146	0.979	0.125	**0.750**	0.646	**0.750**	0.083	0.375	0.208	0.125	**0.500**	**3.021**	1.417	1.104	**1.238**	0.704	0.721
**Charadriformes**	*Charadriidae*	0.000	**0.125**	0.000	0.000	0.000	0.000	0.000	0.000	0.000	0.000	0.000	0.000	0.000	0.000	0.000	0.000	**0.025**	0.000
**Cuculiformes**	*Cuculidae*	**0.063**	0.000	0.000	0.000	0.000	0.000	**0.063**	0.000	0.000	0.000	0.000	0.000	0.000	0.000	0.000	**0.025**	0.000	0.000
**Apodiformes**	*Apodidae*	0.063	0.000	**0.167**	0.000	**0.063**	0.000	0.000	0.000	0.000	0.000	0.000	0.000	0.000	0.000	0.000	0.013	0.013	**0.033**
**Bucerotiformes**	*Upupidae*	**0.292**	0.208	0.083	0.000	**0.563**	0.083	**0.063**	0.000	0.000	0.000	0.000	**0.125**	**0.646**	0.000	0.000	**0.200**	0.154	0.058
**Piciformes**	*Picidae*	0.083	**0.167**	0.000	0.000	**0.125**	0.000	0.000	**0.271**	0.188	0.000	0.000	0.000	0.000	0.000	0.000	0.017	**0.113**	0.038
**Accipitriformes**	*Accipitridae*	0.000	**0.313**	0.000	0.000	**0.083**	0.000	0.000	0.000	**0.063**	0.000	0.000	0.000	0.063	0.000	**0.083**	0.013	**0.079**	0.029
**Coraciiformes**	*Meropidae*	0.000	**5.250**	0.313	0.000	0.000	0.000	0.000	0.000	0.000	0.375	**0.604**	0.000	**0.146**	0.083	0.125	0.104	**1.188**	0.088

	Alcedinidae	0.000	0.000	0.000	0.000	0.000	0.000	0.000	0.000	0.000	0.000	0.000	0.000	0.000	**1.375**	0.000	0.000	**0.275**	0.000
	Subtotal	*0.000*	***5.250***	*0.313*	*0.000*	*0.000*	*0.000*	*0.000*	*0.000*	*0.000*	*0.375*	***0.604***	*0.000*	*0.146*	***1.458***	*0.125*	*0.104*	***1.463***	*0.088*
**Suliformes**	*Phalacrocoracidae*	0.000	0.000	0.000	0.000	0.000	0.000	0.000	0.000	0.000	0.000	0.000	0.000	**0.063**	0.000	0.000	**0.013**	0.000	0.000
**Pelecaniformes**	*Ardeidae*	0.000	0.000	0.000	0.000	0.000	0.000	0.000	0.000	0.000	0.000	0.000	**0.167**	0.000	0.000	0.000	0.000	0.000	**0.033**
**Ciconiidformes**	*Ciconiidae*	0.000	0.000	0.000	0.000	0.000	0.000	0.000	0.000	0.000	0.000	0.000	**0.063**	0.000	0.000	**0.146**	0.000	0.000	**0.042**
**Gruiformes**	*Ralliidae*	0.000	0.000	0.000	0.000	0.000	0.000	0.000	0.000	0.000	0.000	0.000	0.000	**0.333**	0.000	0.000	**0.067**	0.000	0.000
	Gruidae	0.000	0.000	0.000	0.000	0.000	0.000	0.000	0.000	0.000	**2.021**	0.000	0.438	**2.250**	0.000	0.000	**0.854**	0.000	0.088
	Subtotal	*0.000*	*0.000*	*0.000*	*0.000*	*0.000*	*0.000*	*0.000*	*0.000*	*0.000*	***2.021***	*0.000*	*0.438*	***2.583***	*0.000*	*0.000*	***0.921***	*0.000*	*0.088*
**Podicipediformes**	*Podicipediformes*	0.000	0.000	0.000	0.000	0.000	0.000	0.000	0.000	0.000	0.000	0.000	0.000	**0.229**	0.000	0.000	**0.046**	0.000	0.000
**Anseriformes**	*Anatidae*	0.000	0.000	0.000	0.000	0.000	0.000	**0.125**	0.000	0.000	0.000	0.000	0.000	0.000	0.000	0.000	**0.025**	0.000	0.000
**Galliformes**	*Phasianidae*	0.000	0.000	**0.083**	0.000	0.000	0.083	**0.063**	0.000	0.000	0.000	0.000	0.000	0.000	0.000	0.000	0.013	0.000	**0.033**
TOTAL		***12.563***	***15.708***	***5.646***	***19.875***	***16.813***	***11.708***	***9.542***	***5.833***	***10.833***	***15.708***	***4.292***	***9.188***	***123.208***	***170.000***	***10.146***	***36.179***	***42.529***	***9.504***
